# Vascular, inflammatory and perceptual responses to hot water immersion: Impacts of water depth and temperature in young healthy adults

**DOI:** 10.1113/EP092761

**Published:** 2025-07-29

**Authors:** Campbell Menzies, Neil D. Clarke, Charles J. Steward, Charles Douglas Thake, Christopher J. A. Pugh, Tom Cullen

**Affiliations:** ^1^ Centre for Physical Activity, Sport & Exercise Sciences Coventry University Coventry UK; ^2^ School of Life Sciences University of Nottingham Nottingham UK; ^3^ College of Life Sciences, Faculty of Health, Education and Life Sciences Birmingham City University Birmingham UK; ^4^ Cardiff School of Sport & Health Sciences Cardiff Metropolitan University Cardiff UK; ^5^ Centre for Cardiovascular Research, Innovation and Development Cardiff Metropolitan University Cardiff UK

**Keywords:** cortisol, immersion depth, interleukin‐6, passive heating, shear stress, water temperature

## Abstract

Repeated hot water immersion can improve cardiovascular health; however, the respective effects of distinct immersion protocols remain unclear. Twenty‐two healthy adults completed three 30‐min hot water immersion bouts of different water temperatures and immersion depths (40°C shoulder‐deep immersion, 40‐Shoulder; 42°C waist‐deep immersion, 42‐Waist; and 40°C waist‐deep immersion, 40‐Waist) in a randomised crossover design. Vascular, inflammatory and perceptual responses were collected via brachial and superficial femoral artery ultrasound, venous blood sampling and perceptual scales. Rectal temperature increased less in the 40‐Waist (Δ0.5 ± 0.1**°**C) condition than the other conditions (40‐Shoulder: Δ0.9 ± 0.3**°**C, 42‐Waist: Δ0.9 ± 0.3**°**C, *P* < 0.001). Arm skin temperature increased more in the 40‐Shoulder (Δ5.2 ± 1.9**°**C) condition than the other conditions (40‐Waist: Δ2.6 ± 1.0**°**C, 42‐Waist: Δ3.6 ± 1.1**°**C, *P* < 0.001), whilst thigh temperature had a greater increase in the 42‐Waist (8.6 ± 1.3°C) condition than either the 40‐Waist (7.8 ± 0.2**°**C) or 40‐Shoulder (Δ7.8 ± 1.0**°**C) conditions (*P* < 0.001). Brachial artery shear rate was greatest post‐immersion following the 40‐Shoulder condition (40‐Shoulder: Δ121 ± 94/s, 42‐Waist: Δ47 ± 73/s, 40‐Waist: Δ−21 ± 41/s, *P* < 0.001) whereas superficial femoral artery shear rate was largest following the 42‐Waist condition (40‐Shoulder: Δ143 ± 61/s, 42‐Waist: 196 ± 85/s, 40‐Waist: 131 ± 93/s, *P* < 0.001). IL‐6 (*P* = 0.16) and cortisol (*P* = 0.83) responses did not differ between conditions. Perceptual responses were more favourable in the 40‐Waist condition. Taken together, these data demonstrate that the distinct region‐specific arterial responses align with increases in local skin temperature to alterations in hot water immersion protocols, whilst showing that beneficial physiological responses may be accompanied with less favourable perceptual responses.

## Abstract (highlights)

- **What is the central question of this study?**
  How do acute vascular, inflammatory and perceptual responses differ with changes in water temperature, immersion depth and rectal temperature using three different 30‐min hot water immersion protocols?
- **What is the main finding and its importance?**
  Increases in water temperature and immersion depth during 30‐min immersion protocols do not result in different effects on inflammation but have distinct region‐specific arterial haemodynamic effects. Beneficial physiological responses may be accompanied with less favourable perceptual responses demonstrating a potential trade‐off that should be considered during protocol implementation.

## 1 INTRODUCTION

Passive heating, shares many physiological responses with exercise (Cullen et al., 2020), and repeated exposure can result in enhanced cardiorespiratory fitness (Bailey et al., 2016; Hesketh et al., 2019), vascular adaptation (Bailey et al., 2016; Brunt et al., 2016; Carter, Spence, Atkinson, Pugh, Naylor, et al., 2014) and a range of adaptive responses in skeletal muscle (Kim et al., 2020), including angiogenesis (Hesketh et al., 2019), mitochondrial biogenesis (Hafen et al., 2018) and improved glucose metabolism (Ely et al., 2019; Hoekstra et al., 2018). These adaptive processes are underpinned, in part, by the acute shear stress (Carter, Spence, Atkinson, Pugh, Naylor, et al., 2014) and inflammatory (Hoekstra et al., 2018) responses to heating, as well as activation of intracellular signalling pathways, such as increased AMP‐activated protein kinase activity (Liu & Brooks, 2011). Whilst a considerable evidence‐base exists to support the prescription of specific and optimal exercise protocols (American College of Sports Medicine, 2020), in comparison, passive heating research is in its infancy, with little consensus on best practice for heating protocol optimisation in relation to mode, duration, temperature or body coverage. Indeed, duration, temperature and body coverage combine to determine the overall heating dose, making it difficult to isolate their individual effects when interpreting the physiological effects of a heating protocol. Accordingly, the acute physiological responses, such as potential differences in thermo‐physiological, haemodynamic or inflammatory effects between heating protocols are yet to be defined and require systematic investigation to provide valuable insight when designing and implementing novel acute and longitudinal interventions.

Acute increases in blood flow and shear stress are key stimuli for both micro‐ and macro‐vascular chronic adaptation following passive heating (Carter, Spence, Atkinson, Pugh, Cable, et al., 2014, Carter, Spence, Atkinson, Pugh, Naylor, et al., 2014). Indeed, acute changes in shear rate and blood pressure after a single exercise or hot water immersion exposure positively correlated with subsequent adaptation to repeated usage (Dawson et al., 2018; Roxburgh et al., 2023). Local limb temperature is a key driver for increased peripheral blood flow to that limb (Chiesa et al., 2016; Koch Esteves et al., 2024; Watanabe et al., 2024); however, whole body heating can increase limb temperature and blood flow in unheated limbs (Chaseling et al., 2023; Heinonen et al., 2011). Indeed, lower body heating can evoke systemic effects, as evidenced by increased brachial artery shear rate and flow‐mediated dilatation (Amin et al., 2021; Cheng et al., 2021). These systemic effects may be triggered by whole body thermoregulatory responses that occur following increases in core body temperature (Low et al., 2011). Accordingly, although acute arterial haemodynamic responses appear to be evoked by increases in both local and core body temperature, the magnitude of response varies across vascular beds (Cheng et al., 2021; Francisco et al., 2021; Hoekstra et al., 2021) and is likely significantly influenced by the body coverage of the heating stimulus. Moreover, blood flow continues to increase when rectal temperature is clamped during prolonged hot water immersion (Francisco et al., 2021). This means that studies investigating the effects of rectal temperature on haemodynamic responses during passive heating are likely confounded by duration (e.g., Chiesa et al., 2016). Manipulation of heating dose through different water temperatures and immersion depths may allow for duration and rectal temperature‐matched protocols to be assessed to elucidate systemic and localised haemodynamic responses.

Similar to arterial haemodynamic responses, many inflammatory and endocrine responses appear sensitive to increases in core body temperature (Rhind et al., 2004). Acute elevations in inflammatory cytokines, such as interleukin‐6 (IL‐6), are suggested to be important in the subsequent anti‐inflammatory response and protecting against chronic inflammatory and metabolic diseases (Nash et al., 2023). Although mixed findings of acute IL‐6 responses to passive heating have been observed, with some (Hoekstra et al., 2020, 2021; Mansfield et al., 2021) but not all (Gibson et al., 2025; Monroe et al., 2021) studies showing an acute elevated IL‐6 concentration, it has recently been suggested that IL‐6 may increase following passive heating in a dose‐dependent manner (Hoekstra et al., 2020). However, the specific factors that influence ‘dose’ are currently ill‐defined. Heat‐induced IL‐6 release from skeletal muscle has been demonstrated *ex vivo* (Obi et al., 2017; Welc et al., 2012), suggesting that increases in muscle rather than core temperature may be the primary stimulus for the acute inflammatory response to passive heating. Indeed, human studies have shown that IL‐6 responses to local and whole‐body heating appear similar despite smaller increases in rectal temperature with local heating (Hoekstra et al., 2021). Therefore, the potential inflammatory responses to passive heating may differ with heating duration, body coverage, or temperature but this requires further investigation using carefully controlled experimental protocols, with systematic manipulation of whole body and local temperature, to enable the relevant stimuli to be isolated.

Whilst passive heating may offer physiological benefits, some protocols can be uncomfortable, difficult to tolerate and result in negative effects, such as orthostatic hypotension (Steward et al., 2023). Mitigation strategies may be effective in reducing these effects (Steward et al., 2023); however, they may also reduce the desired physiological responses. Indeed, upper body cooling with lower body heating improves perceptual responses compared to whole body heating, but at the cost of attenuated increases in inflammatory and haemodynamic responses (Hoekstra et al., 2021). Accordingly, the balance between the desired physiological responses and the tolerability or potential negative effects is an important consideration when prescribing any passive heating intervention.

The present study aimed to systematically manipulate water temperature and immersion depth to investigate limb‐specific vascular, acute inflammatory, and perceptual responses to hot water immersion protocols of matched‐duration and eliciting similar increases in rectal temperature. It was hypothesised that: (i) all immersion protocols would increase arterial shear rate in both submerged and non‐submerged limbs, with higher water temperatures augmenting these responses, and the greatest increase being observed in the local arteries of submerged limbs; (ii) the IL‐6 response would be greater with larger increases in rectal temperature; and (iii) thermal comfort, desired exit time and dizziness upon standing would differ between conditions.

## 2 METHODS

### 2.1 Participants

Based on previously observed effects for acute vascular responses (*d *= 0.74; Chaseling et al., 2023) and IL‐6 (*d *= 0.65; Mansfield et al., 2021) between different heating protocols, the present study was powered a priori to detect an effect size of *d *= 0.65, with an α of 0.05 and an 80% power resulting in a required sample size of 22. Accordingly, 22 healthy adults (13 males: 29 ± 6 years, 80.3 ± 14.4 kg, 1.78 ± 0.09 m, 25.3 ± 4.4 kg/m^2^. Nine females: 27 ± 6 years, 62.1 ± 8.8 kg, 1.64 ± 0.05 m, 22.9 ± 2.2 kg/m^2^) were briefed and provided written consent to participate in the present study. Ethical approval was provided by the Coventry University Ethics committee (P146084) and conformed to the *Declaration of Helsinki*, except for prior registration in a database. Participants were aged 18–40 years old, non‐smokers, with no history of cancer or metabolic illness, and had not been exposed to regular passive heating for at least 3 months prior to participating in the study.

### 2.2 Experimental design

Participants attended the laboratory (20.0 ± 1.4°C, 53 ± 10% relative humidity) on three occasions to complete 30 min of hot water immersion. The three experimental conditions consisted of (i) 40°C shoulder‐deep immersion (40‐Shoulder) (40.0 ± 0.1°C), (ii) 42°C waist‐deep immersion (42‐Waist) (42.0 ± 0.1°C) or (iii) 40°C waist‐deep immersion (40‐Waist) (40.0 ± 0.1°C) and were completed in a randomised crossover design. These conditions were chosen such that the 40‐Shoulder and 42‐Waist conditions elicited similar increases in rectal temperature based on previous research (Hoekstra et al., 2018; Mansfield et al., 2021) and pilot testing within our laboratory. A 30‐min duration was chosen as the shortest duration previously shown to elicit adaptation with repeated hot water immersion exposures (Bailey et al., 2016). For the waist‐deep conditions, participants sat on an 18 cm stool without immersion of their arms in a water depth of ∼36 cm. In the shoulder‐deep condition participants sat on the bottom of the hot tub with their arms submerged in a water depth of 47–52 cm, adjusted according to the participants’ height, and moved to the stool for ∼3 min with non‐submersion of their arms after 5, 15 and 25 min for the assessment of blood pressure and vascular imaging. Water temperature was constantly monitored using a thermistor (Grant Instruments, Cambridge, UK) and maintained using a hot tub heating generator (LazySpa Majorca, Bestway International Ltd, Hong Kong) set to 40°C, with addition and removal of hot or cold water as required to maintain temperature and depth.

Visits were separated by a minimum of 48 h and conducted at the same time of day (±2 h), with the order of experimental conditions determined by a randomisation sequence in Excel. To control for effects of the menstrual cycle on thermoregulatory and endothelial responses (Charkoudian et al., 2017; Hashimoto et al., 1995), sessions were completed between Days 1 and 7 after their self‐reported onset of menses for eumenorrhoeic participants (*n* = 6), or during the seven inactive days for participants taking oral contraceptive pills (*n* = 2), with one participant having an intra‐uterine device and no menstrual cycle.

### 2.3 Experimental protocol

**FIGURE 1:**
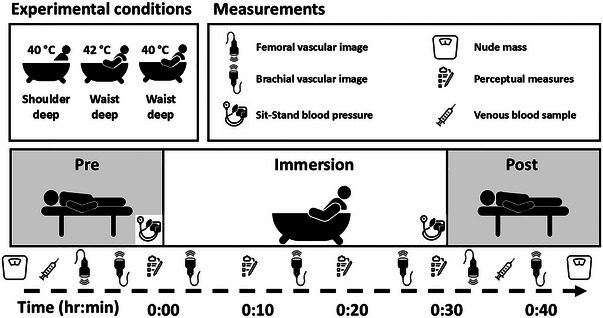
Schematic diagram of experimental design representing the three experimental conditions, time course of immersion and measurements taken.

Prior to arrival in the laboratory, participants were instructed to refrain from strenuous exercise for 24 h, be fasted for >6 h and refrain from caffeine consumption for 12 h. Upon arrival, participants commenced with the experimental protocol (Figure 1).

### 2.4 Thermo‐physiological measures

Rectal temperature was monitored at 5‐min intervals throughout using a rectal thermometer (Grant Instruments) self‐inserted 10 cm beyond the anal sphincter. Heart rate was recorded using a heart rate chest strap (H10, Polar, Kempele, Finland). Nude mass (Seca; Bodycare, Birmingham, UK) was recorded before and after the experimental protocol to estimate whole body sweat rates. In a subset of six participants (five males, one female), skin temperature was measured (DS1921H iButton, Maximum, Dallas Semiconductor Corp., Dallas, TX, USA) on the upper arm and thigh adjacent to the location of the probe location for vascular imaging.

### 2.5 Perceptual measures

Thermal sensation and thermal comfort were measured on scales ranging from +5 (hot and very comfortable, respectively) to −5 (cold and very uncomfortable, respectively) modified from Epstein & Moran (2006), with thermal desirability measured from +3 (a significantly lower temperature would be better) to −3 (a significantly higher temperature would be better) modified from Oi et al. (2012). During immersion, participants were asked for ratings of thermal sensation for above and below the water separately, thermal comfort overall and thermal desirability of the water. Symptoms of dizziness, confusion, nausea, tiredness and headaches were monitored on a 0–10 scale, with the scale anchored at 0 – no symptoms, 3 – mild symptoms, 5 – moderate symptoms, 7 – severe symptoms and 10 – have to stop (Coris et al., 2006). Participants were also asked every 5 min whether under conditions outside of the laboratory they would choose to exit the water. Following the completion of all the conditions, participants ranked the three conditions in order of favourite to least favourite.

### 2.6 Orthostatic hypotension

Orthostatic hypotension was measured as previously described (Steward et al., 2023) and defined according to clinical guidelines (Freeman et al., 2011) as a reduction of ≥20 mmHg in systolic blood pressure (SBP) or ≥10 mmHg in diastolic blood pressure (DBP) upon standing when compared with values obtained at baseline, before the immersion period. Briefly, blood pressure (M3 Blood pressure monitor, Omron, Kyoto, Japan) was recorded in duplicate whilst seated on a chair at baseline following a 5‐min period of quiet rest. Upon standing, participants reported symptoms of dizziness on the same 0–10 scale used for symptoms of heat illness (Coris et al., 2006) whilst a single blood pressure measurement was taken. Participants were instructed to stand up gradually and give a rolling dizziness score (i.e., repeated verbalisation of perceived dizziness every few seconds) with maximum dizziness recorded. Similarly, maximum heart rate upon standing was recorded due to the role of heart rate in maintaining cardiac output to counteract reductions in blood pressure during orthostasis (Convertino, 2014).

### 2.7 Vascular imaging

The superficial femoral artery was imaged pre‐ and 3 min post‐immersion. Brachial artery imaging was performed pre‐ and 9 min post‐immersion. Resting images were performed following at least 10 min of supine rest. During immersion, brachial artery diameter, blood flow and shear rate were measured after 5, 15 and 25 min, whilst participants rested their arm on the edge of the hot tub. Vascular imaging was performed using a 15‐MHz multifrequency linear array probe attached to a high‐resolution duplex ultrasound machine (T3300; Tersaon, Burlington, MA, USA). Images were taken following optimisation of the longitudinal B‐mode image of the lumen‐arterial interface, with simultaneous Doppler velocity assessments collected using the smallest possible insonation angle (always <60°). For each visit, once optimal image acquisition was achieved, and the probe location was marked on the skin to ensure imaging of the same arterial section.

Analysis of arterial diameter, blood flow and shear rate was performed using custom‐designed edge‐detection and wall‐tracking software, as previously described elsewhere (Woodman et al., 2001). This semi‐automated software is independent of investigator bias and has greater reproducibility than manual methods (Woodman et al., 2001), and in the present study had a between visit coefficient of variation (CV) at rest of 3.9 ± 3.3% and 4.7 ± 3.5% for brachial and superficial femoral artery diameter, respectively. Blood flow was calculated as lumen cross‐sectional area multiplied by Doppler velocity, with shear rate (as an estimate of shear stress without viscosity) calculated as four times the mean blood velocity/vessel diameter.

### 2.8 Blood sampling and analysis

Venous blood samples (∼10 mL) were obtained pre‐ and ∼5 min post‐immersion through repeated venepuncture. Blood samples were split for the determination of haematocrit and haemoglobin (Hb 201+ System, Haemocue, Ängelholm, Sweden), with the remaining blood spun at 3000 *g* for 10 min in an EDTA vacutainer before aliquoting the plasma, which was stored at −80°C until later analysis. IL‐6 and cortisol were analysed using commercially available ELISA kits (D6050 and KGE008B, respectively; R&D Systems, Abingdon, UK). Samples were diluted (DY997, R&D Systems) in a ratio of 1:60 for the cortisol assay to ensure concentrations were within the dynamic range of the assay. Concentrations were determined using interpolation of a four‐parameter standard curve (GraphPad Prism, GraphPad Software, Boston, MA, USA) before being adjusted for changes in plasma volume calculated according to Dill and Costill (1974). To minimise variation between assays, all samples from an individual participant were analysed in the same assay and in the present study, and the intra‐assay CVs for these assays were 8.8 ± 8.1% and 3.4 ± 2.8% for IL‐6 and cortisol, respectively. Due to sampling issues, blood samples were not obtained for two participants (one male, one female), resulting in a sample of *n* = 20 for these outcomes.

### 2.9 Statistical analysis

Data are reported as means ± standard deviation, or median (lower quartile, upper quartile) unless stated otherwise, with mean difference and 95% confidence intervals (95% CI) presented as mean difference [lower limit, upper limit] when describing differences between conditions. Analysis was conducted, unless otherwise stated, in RStudio using the functions and packages stated below, with an example of the code found in Supporting information. Statistical significance was accepted as α < 0.05. Perceptual data (Thermal comfort, Thermal sensation, Thermal desirability, Condition preference) were examined within each condition for an effect of time using the *freidman.test* function from the *stats* package to conduct Friedman's test. Differences between conditions were also analysed using Friedman's test and where differences were detected, *post hoc* testing was performed using the *pairwise.wilcox.test* function from the *stats* package, with Bonferroni adjustments for multiple comparisons. Count data (desired exit time, presence of orthostatic hypotension) were analysed using the *chisq.test* and *chisq.post.hoc* functions from the *stats* and *fifer* packages, respectively, to perform chi‐squared tests with Fisher's exact tests applied where significant effects of condition were found. Change in nude mass and plasma volume were analysed using a one‐way repeated measures ANOVA, whilst all other data were examined using a two‐way (Time × Condition) repeated measures ANOVA using the *anova_test* function from the *Rstatix* package, with effect sizes for main effects presented as partial eta squared (η_p_^2^). Violations of normality were not assessed due to the risks associated with non‐Gaussian models and the robustness of ANOVA in violations of this assumption (Knief & Forstmeier, 2020). Where an interaction effect was identified, *post hoc* analysis between conditions at the separate time points was performed using the *pairwise_t_test* function from the *Rstatix* package, with Bonferroni adjustments for multiple comparisons. Although not the focus of this study, sex differences were analysed by adding Sex as a between‐subjects variable into a three‐way (Sex × Time × Condition) ANOVA, with the results available in Supporting information.

## 3 RESULTS

### 3.1 Thermo‐physiological measures

**FIGURE 2:**
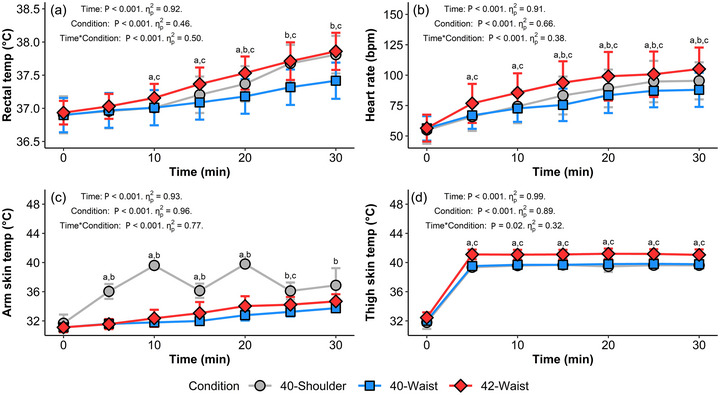
Mean thermo‐physiological responses to each condition over time. (a) Rectal temperature, (b) heart rate, (c) arm skin temperature and (d) thigh skin temperature. Significance is denoted as follows: ^a^40‐Shoulder vs. 42‐Waist, ^b^40‐Shoulder vs. 40‐Waist and ^c^42‐Waist vs. 40‐Waist.

Rectal temperature increased in all conditions but was lower at the end of immersion in the 40‐Waist condition compared to both the 40‐Shoulder (mean difference: 0.4 [0.3, 0.5]°C, *P* < 0.001) and 42‐Waist (mean difference: 0.4 [0.4, 0.5]°C, *P* < 0.001) conditions, which were similar (mean difference: 0.1°C [−0.2, 0.1]°C, *P* = 1.0) (Figure 2a). Similarly, changes in nude mass were not different between 40‐Shoulder (Δ−0.4 ± 0.2 kg) and 42‐Waist (Δ−0.4 ± 0.2 kg, *P* = 0.70), but with a smaller reduction in 40‐Waist (Δ−0.3 ± 0.2 kg mean difference vs. 40‐Shoulder: −0.5 [−0.6, −0.4] kg, *P* < 0.001; mean difference versus 42‐Waist: −0.4 [−0.5, −0.4] kg, *P* = 0.003) resulting in a significant main effect of condition (*P* < 0.001. η_p_^2^ = 0.40). In contrast, heart rate was higher at the end of immersion in the 42‐Waist condition than the 40‐Shoulder condition (mean difference: 10 [5, 14] bpm, *P* = 0.001), which was in turn higher than the 40‐Waist condition (mean difference: 7 bpm [3, 12] bpm, *P* = 0.003) (Figure 2b). Arm skin temperature increased during immersion and was greater in the 40‐Shoulder condition compared to the 40‐Waist condition throughout immersion and compared to the 42‐Waist for the first 20 min of immersion (Figure 2c). Arm skin temperature was also higher in the 42‐Waist condition, compared to the 40‐Waist condition after 25 min. Thigh skin temperature increased during immersion and was greater in the 42‐Waist condition than the 40‐Shoulder or 40‐Waist condition, which were similar (Figure 2d).

### 3.2 Blood pressure responses

SBP and DBP reduced during immersion in all conditions, with the greatest reduction in systolic blood pressure, but the smallest reduction in DBP was observed in the 40‐Waist condition (Table 1). There was no difference in SBP between conditions post‐immersion; however, DBP was different between all conditions, being lowest in the 40‐Shoulder condition (mean difference vs. 40‐Waist: −9 [−12, −6] mmHg, *P* < 0.001; mean difference vs. 42‐Waist: −4 [−7, −1] mmHg, *P* = 0.04), and highest in the 40‐Waist condition (mean difference vs. 42‐Waist: 5 [2, 8] mmHg, *P* = 0.02).

**TABLE 1:** Summary of mean antegrade and retrograde responses in the brachial and superficial femoral arteries, and mean blood pressure for each condition over time.

Variable​	Condition​	Pre​	5 min​	15 min​	25 min​	Post​	Statistics
Systolic blood pressure (mmHg)	40‐Shoulder​	118 ± 10	109 ± 13^b^​	112 ± 14	113 ± 12	123 ± 10	Time: *P* < 0.001, η_p_^2^ = 0.66 Condition: *P* = 0.60, η_p_^2^ = 0.13 Time × Condition: *P* = 0.02, η_p_^2^ = 0.10
	42‐Waist​	118 ± 8	107 ± 14	112 ± 14	114 ± 14^c^	124 ± 8	
	40‐Waist​	119 ± 11	105 ± 14^b^​	107 ± 17	108 ± 17^c^​	123 ± 111	
Diastolic blood pressure (mmHg)	40‐Shoulder​	69 ± 6	61 ± 8	56 ± 10	54 ± 7^b^​	58 ± 9^a,b^​	Time: *P* < 0.001, η_p_^2^ = 0.68 Condition: *P* = 0.003, η_p_^2^ = 0.25 Time × Condition: *P* < 0.001, η_p_^2^ = 0.16
	42‐Waist​	70 ± 5	60 ± 9	58 ± 10	57 ± 8	62 ± 9^a,c^​	
	40‐Waist​	70 ± 6	59 ± 7	61 ± 9	58 ± 9^b^​	67 ± 6^b,c^​	
Femoral antegrade shear rate (/s)	40‐Shoulder​	77 ± 26​	—	—	—	196 ± 60^a^​	Time: *P* < 0.001, η_p_^2^ = 0.83 Condition: *P* < 0.001, η_p_^2^ = 0.33 Time × Condition: *P* < 0.001, η_p_^2^ = 0.29
	42‐Waist​	84 ± 33​	—	—	—	255 ± 95^a,c^​	
	40‐Waist​	92 ± 56​	—	—	—	200 ± 85^c^​	
Femoral retrograde shear rate (/s)​	40‐Shoulder​	−30 ± 12​	—	—	—	−5 ± 7​	Time: *P* < 0.001, η_p_^2^ = 0.81 Condition: *P* = 0.60, η_p_^2^ = 0.02 Time × Condition: *P* = 0.48, η_p_^2^ = 0.03
	42‐Waist​	−28 ± 12​	—	—	—	−3 ± 7​	
	40‐Waist​	−28 ± 15​	—	—	—	−6 ± 7​	
Brachial antegrade​ shear rate (/s)​​	40‐Shoulder​	59 ± 35​	184 ± 69^b^​	395 ± 143^a,b^​	457 ± 139^a,b^​	165 ± 85^a,b^​	Time: *P* < 0.001, η_p_^2^ = 0.87 Condition: *P* < 0.001, η_p_^2^ = 0.66 Time × Condition: *P* < 0.001, η_p_^2^ = 0.38
	42‐Waist​	64 ± 38​	167 ± 76​	276 ± 94^a,c^​	376 ± 121^a,c​^	107 ± 66^a,c^​	
	40‐Waist​	68 ± 41​	140 ± 76^b​^	201 ± 73^b,c^​	275 ± 102^b,c​^	51 ± 29^b,c^​	
Brachial retrograde​ shear rate (/s)​	40‐Shoulder​	−16 ± 14​	−16 ± 20^a,b ^​	−1 ± 5^a,b^​	−2 ± 4^b​^	0 ± 1^a,b^​	Time: *P* < 0.001, η_p_^2^ = 0.36 Condition: *P* < 0.001, η_p_^2^ = 0.41 Time × Condition: *P* < 0.001, η_p_^2^ = 0.16
	42‐Waist​	−12 ± 10​	−34 ± 32^a^​	−12 ± 13^a^​	−9 ± 20​	−8 ± 9^a,c​^	
	40‐Waist​	−13 ± 10​	−34 ± 29^b^​	−28 ± 36^b^​	−13 ± 21^b^​	−17 ± 13^b,c​^	

*Note*: Participants were supine for pre‐ and post‐measurements but sat upright during immersion at 5, 15 and 25 min. Significance is denoted as follows: ^a^40‐Shoulder vs. 42‐Waist, ^b^40‐Shoulder vs. 40‐Waist and ^c^42‐Waist vs. 40‐Waist.

### 3.3 Superficial femoral artery responses

**FIGURE 3:**
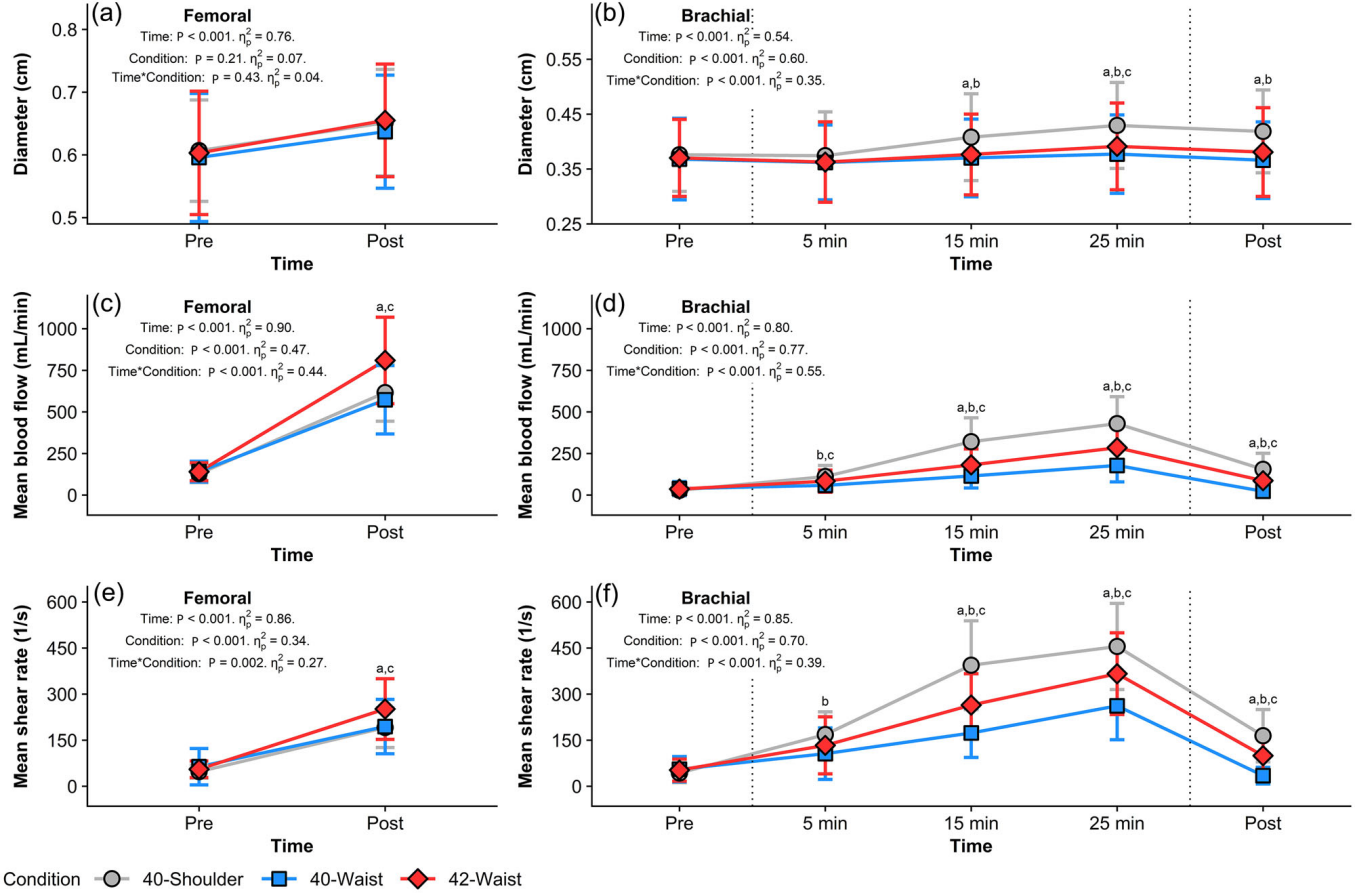
Mean arterial diameter, mean blood flow and mean shear rate responses across time for each condition for the superficial femoral (a, c, e) and brachial (b, d, f) arteries, respectively. The data between the vertical dotted lines in (b, d, f) were obtained from the brachial artery during immersion, whilst participants were sitting upright, whilst pre‐ and post‐measures across all panels were obtained with participants supine. Significance is denoted as follows: ^a^40‐Shoulder vs. 42‐Waist, ^b^40‐Shoulder vs. 40‐Waist and ^c^42‐Waist vs. 40‐Waist.

There was a similar increase in diameter in all conditions (Figure 3a). However, there was a difference between conditions for mean blood flow (Figure 3c), with greater values observed post‐immersion in the 42‐Waist condition than either the 40‐Shoulder (mean difference: 193 [109, 278] mL/min, *P* < 0.001) or 40‐Waist (mean difference: 273 [154, 319] mL/min, *P* < 0.001) condition. These differences were also observed post‐immersion for mean shear rate (Figure 3e; mean difference 42‐Waist vs. 40‐Shoulder: 60 [30, 91]/s, *P* = 0.003; 42‐Waist vs. 40‐Waist: 57 [35, 79]/s, *P* < 0.001) and antegrade shear rate (Table 1; mean difference 42‐Waist vs. 40‐Shoulder: 59 [29, 89]/s, *P* = 0.003; 42‐Waist vs. 40‐Waist: 55 [33, 76]/s, *P* < 0.001). There were no observed differences in these variables between the 40‐Shoulder and 40‐Waist conditions. In contrast, there was no difference between conditions in the significant reduction of retrograde shear rate observed following immersion (Table 1).

### 3.4 Brachial artery responses

Diameter increased during immersion peaking at 25 min before falling post‐immersion (Figure 3b), with larger values observed at 25 min in the 40‐Shoulder condition than either the 42‐Waist (mean difference: 0.38 [0.27, 0.50] cm, *P* < 0.01) or 40‐Waist (mean difference: 0.52 [0.40, 0.64] cm, *P* < 0.01) condition, which also differed (mean difference: 0.14 [0.04, 0.24] cm, *P* = 0.05). Similar responses were observed, with the largest values observed at 25 min, for mean blood flow (Figure 3d; mean differences: 40‐Shoulder vs. 42‐Waist: 145 [97, 193] mL/min, *P* < 0.001; 40‐Shoulder vs. 40‐Waist: 251 [199, 303] mL/min, *P* < 0.001; 42‐Waist vs. 40‐Waist: 106 [72, 141] mL/min, *P* < 0.001), mean shear rate (Figure 3f; mean differences: 40‐Shoulder vs. 42‐Waist: 89 [34, 143]/s, *P* = 0.01; 40‐Shoulder vs. 40‐Waist: 193 [134, 253], *P* < 0.001; 42‐Waist vs. 40‐Waist: 105 [57, 153]/s, *P* = 0.001), and antegrade shear rate (Figure 3d; mean differences: 40‐Shoulder vs. 42‐Waist: 81 [32, 129]/s, *P* = 0.01; 40‐Shoulder vs. 40‐Waist: 182 [125, 238]/s, *P* < 0.001; 42‐Waist vs. 40‐Waist: 101 [53, 149]/s, *P* = 0.001). Retrograde shear rate was different between all conditions post‐immersion (Table 1) being negligible in the 40‐Shoulder condition and highest in the 40‐Waist condition (mean differences: 40‐Shoulder vs. 42‐Waist: 8 [4, 11]/s, *P* < 0.001; 40‐Shoulder vs. 40‐Waist: 16 [11, 22]/s, *P* < 0.001; 42‐Waist vs. 40‐Waist: 9 [3, 14]/s, *P* = 0.02).

### 3.5 Venous blood measures

**FIGURE 4:**
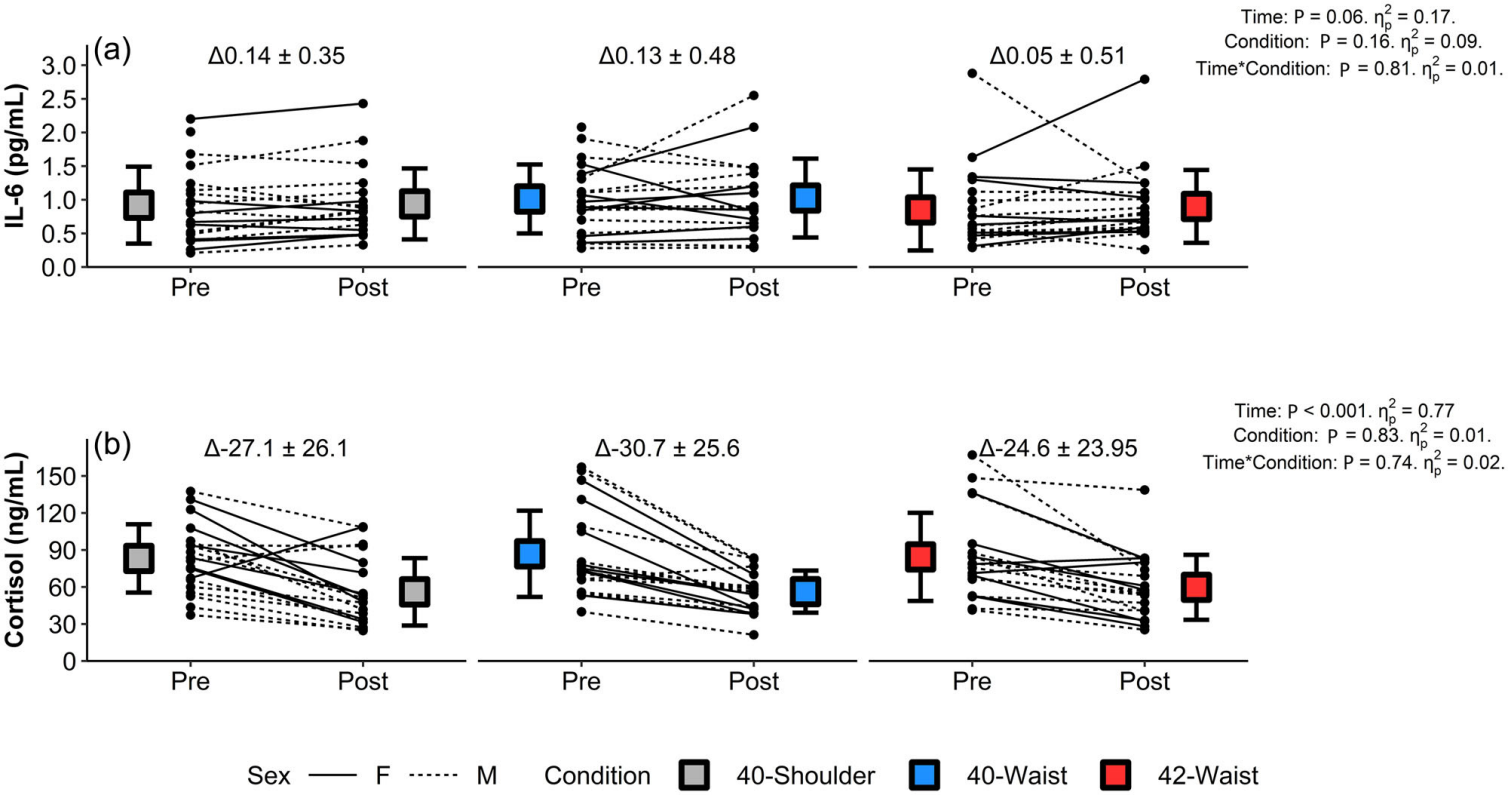
Mean plasma IL‐6 (a) and cortisol (b) concentrations pre‐ and post‐immersion for each condition. Connected lines and smaller dots represent individual responses for each participant. IL‐6, interleukin‐6.

Increases in plasma volume were observed following each bout of immersion and were similar between all conditions (40‐Shoulder: Δ11 ± 16%, 42‐Waist: Δ9 ± 12%, 40‐Waist: Δ9 ± 10%, *P* = 0.80, η_p_^2^ = 0.01). IL‐6 did not differ over time or between conditions (Figure 4a), whilst cortisol reduced post‐immersion, but did not differ between conditions (Figure 4b).

### 3.6 Perceptual measures

Summary data for all perceptual measures are shown in Table 2. Thermal sensation (above and below the water), thermal comfort and thermal desirability were similar between 40‐Shoulder and 42‐Waist for 20 min but differed at 30 min where participants felt hotter, less comfortable and desired a lower water temperature in the 42‐Waist condition. In the 40‐Waist condition, thermal sensation (above the water), thermal comfort, and thermal desirability were different from the other two conditions for the first 20 min of immersion with participants feeling cooler, more comfortable and having less desire to reduce the water temperature. However, these variables were similar to the 40‐Shoulder condition after 30 min, when participants in this condition had moved to the stool and removed their arms from the water. Symptoms of heat illness were not largely prevalent in any condition (Table 3). After 20 min of immersion, there was a difference between conditions for the number of participants who had indicated they would choose to exit the water outside of laboratory conditions (40‐Shoulder: *n* = 12, 42‐Waist: *n* = 13, 40‐Waist: *n* = 5, *P* = 0.03); however, comparisons between conditions were not identified with *post hoc* testing. A further three participants in each condition indicated they would exit the water prior to the 30‐min time point and did not alter the statistical comparison between conditions. There was no clear difference in participant preference for each protocol (*P* = 0.11). However, 40‐Shoulder was rated as the favourite condition by 13 participants, compared to seven in the 40‐Waist condition and two in the 42‐Waist condition. Inversely, six participants rated 40‐Shoulder as their least favourite condition, which was fewer than both the 42‐Waist (*n* = 9) and 40‐Waist (*n* = 7) conditions.

**TABLE 2:** Median perceptual responses over time for each condition.

Variable	Condition	0	10	20	30
Thermal sensation (below water) (a.u)	40‐Shoulder^*^	−1 (−2.75, −0.25)	3 (1.25, 3)	4 (3, 4)^b^	4 (2, 4)
	42‐Waist^*^	−1 (−2, 0)	3 (2, 4)^c^	4 (3.25, 4)^b,c^	4 (4, 5)^c^
	40‐Waist^*^	−1 (−2, −0.25)	2 (1, 3)^c^	3 (2, 3)^c^	3 (3, 4)^c^
Thermal sensation (above water) (a.u)	40‐Shoulder^*^	−1 (−2.75, −0.25)	1 (0.25, 2)^b^	3 (1.25, 3)^b^	2 (1, 3)^a^
	42‐Waist^*^	−1 (−2, 0)	1 (0, 2)^c^	3 (2, 3)^c^	4 (3, 4.75)^a,c^
	40‐Waist^*^	−1 (−2, −0.25)	0 (−1, 0)^b,c^	0.5 (0, 1)^b,c^	1 (1, 2)^c^
Thermal comfort (a.u)	40‐Shoulder^*^	0 (−1, 0.75)	2 (0, 3)	−1 (−2, 1)^b^	0 (−1.75, 1.75)^a^
	42‐Waist^*^	0 (−1, 1.75)	0.5 (−1, 2)	−1 (0, −2)^c^	−2 (−4, −1)^a,c^
	40‐Waist^*^	0 (−1, 0.75)	2 (1, 3)	2 (0, 2.75)^b,c^	0 (−1, 1)^c^
Thermal desirability (of the water) (a.u)	40‐Shoulder^*^	—	0 (0, 0)^b^	1 (0, 1)^b^	1 (0, 1)^a^
	42‐Waist^*^	—	0 (0, 1)^c^	1 (1, 2)^c^	2 (1, 2)^a,c^
	40‐Waist^*^	—	0 (−0.75, 0)^b,c^	0 (0, 0)^b,c^	0.5 (0, 1)^c^

*Note*: Significance is denoted as follows: ^*^significant effect of time within variable, ^a^40‐Shoulder vs. 42‐Waist, ^b^40‐Shoulder vs. 40‐Waist and ^c^42‐Waist vs. 40‐Waist.

**TABLE 3:** Frequency of symptoms of heat illness reported in each condition.

Condition	Confusion	Dizziness	Headaches	Nausea	Tiredness	Total
40‐Shoulder	22/0/0/0/0	21/0/0/1/0	22/0/0/0/0	22/0/0/0/0	21/0/1/0/0	108/0/1/1/0
42‐Waist	22/0/0/0/0	20/1/1/0/0	20/2/0/0/0	22/0/0/0/0	20/1/1//0	104/2/4/0/0
40‐Waist	22/0/0/0/0	22/0/0/0/0	22/0/0/0/0	22/0/0/0/0	20/1/1//0	108/1/1/0/0

*Note*: Values are shown as the peak response across the protocol across all participants for scores of 0/1–2/3–4/5–6/7–10.

### 3.7 Orthostatic hypotension

**FIGURE 5:**
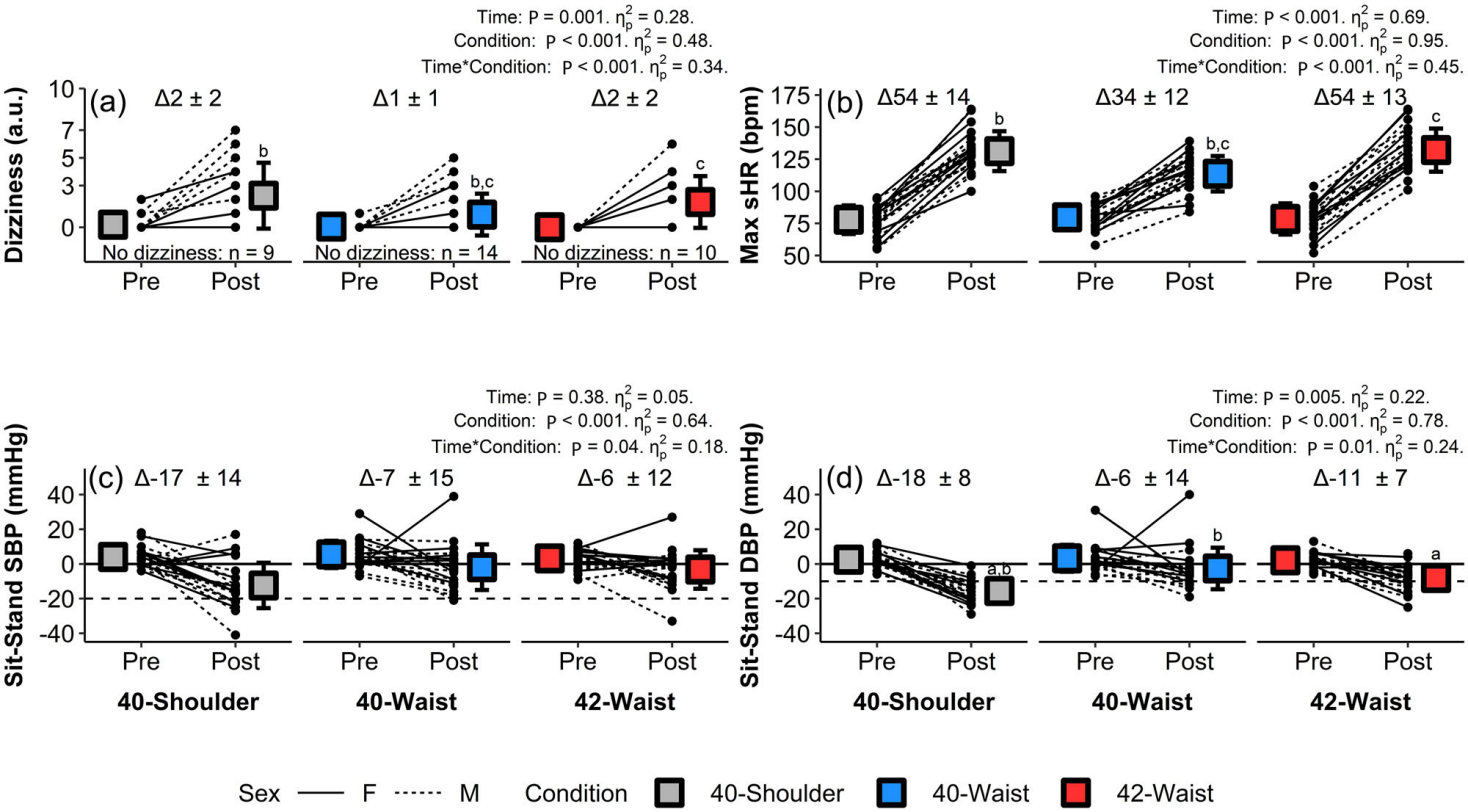
Mean responses upon standing pre‐ and post‐immersion for each condition. (a) Standing dizziness, (b) maximum heart rate upon standing, (c) Sit‐Stand SBP and (d) Sit‐Stand DBP. Significance is denoted as follows: ^a^40‐Shoulder vs. 42‐Waist, ^b^40‐Shoulder vs. 40‐Waist and ^c^42‐Waist vs. 40‐Waist. Connected lines and smaller dots represent individual responses for each participant. Dashed horizontal lines represent the clinical threshold for orthostatic hypotension in (c) SBP (−20 mmHg), and (d) DBP (−10 mmHg). DBP, diastolic blood pressure; max sHR, maximum heart rate upon standing; SBP, systolic blood pressure.

The prevalence of orthostatic hypotension was significantly different between conditions (*P* = 0.001), with a higher prevalence in the 40‐Shoulder condition (17/22, 77%) than the 40‐Waist condition (5/22, 23%, *P* = 0.002), but no statistical difference with the 42‐Waist condition (9/22, 41%, *P* = 0.09). These differences were largely driven by changes in Sit‐Stand DBP (Figure 5d) rather than SBP (Figure 5c), with lower values observed post‐immersion in the 40‐Shoulder condition than either the 42‐Waist (mean difference: −7 [−3, −10] mmHg, *P* = 0.001) or 40‐Waist (mean difference: −13 [−7, −19] mmHg, *P* = 0.004) condition. Lower values of dizziness upon standing were observed post‐immersion in the 40‐Waist condition than in the 40‐Shoulder (mean difference: −1 [−1, −2] a.u., *P* = 0.008) and 42‐Waist (mean difference: −1 [0, −1] a.u., *P* = 0.01) Figure 5a) conditions. Maximum heart rate upon standing displayed a similar pattern with an increase in heart rate following all immersion conditions, but lower values post‐immersion in the 40‐Waist condition than the 40‐Shoulder (mean difference: −18 [−12, −23] bpm, *P* < 0.001) or 42‐Waist (mean difference: −18 [−14, −23] bpm, *P* < 0.001) (Figure 5b) condition.

## 4 DISCUSSION

The present study aimed to investigate acute arterial haemodynamic, inflammatory and perceptual responses following three different 30‐min hot water immersion protocols, which manipulated water temperature, immersion depth and rectal temperature to isolate their effects. The main findings were the following. (i) Limb submersion results in the greatest increases in arterial shear (i.e., greater increases in the brachial artery with 40‐Shoulder than 42‐Waist, despite similar increases in rectal temperature). However, higher water temperatures augment the response in both submerged (i.e., femoral artery responses between 42‐Waist and 40‐Waist) and non‐submerged limbs (i.e., brachial artery responses between 42‐Waist and 40‐Waist). Increased shear rate was accompanied by greater elevations in local skin temperature highlighting the importance of local temperature in arterial haemodynamic responses. (ii) Thirty minutes of hot water immersion was insufficient to elicit significant changes in circulating IL‐6 concentrations. In contrast, cortisol concentrations reduced following immersion but did not differ between conditions. (iii) Increasing the heating stimulus through either immersion depth or water temperature increases symptoms of dizziness and maximum heart rate, whilst reducing the decrease in DBP upon standing following immersion and decreasing thermal comfort during immersion.

### 4.1 Vascular responses

The present study provides novel insight into the acute effects of hot water immersion, demonstrating that shear rate responses are most pronounced within submerged limbs, and that higher water temperatures augment this response. Higher thigh skin temperature corresponded to increased shear rate in the superficial femoral artery, while increased arm skin temperature was accompanied by elevated brachial artery shear rate. In contrast, despite similar increases in rectal temperature between the 40‐Shoulder and 42‐Waist conditions, increases in shear rate were both protocol and limb‐specific, with increases in rectal temperature having no effect on superficial femoral artery shear rate when thigh skin temperatures were similar between the 40‐Shoulder and 40‐Waist conditions. These findings support the importance of local temperature on arterial haemodynamic responses to heating (Chiesa et al., 2016; Heinonen et al., 2011; Koch Esteves et al., 2024; Watanabe et al., 2024) and emphasise that elevations in core body temperature are not the primary driver of vascular responses. Previous data have demonstrated sequentially greater increases in rectal temperature (in response to a single prolonged heating bout) result in progressive increases in superficial femoral artery shear rate, with a ∼30/s increase in shear rate when comparing an increase in rectal temperature of 0.5°C and 1.0°C (Chiesa et al., 2016). However, shear rate continues to increase by ∼100/s across 20 min when rectal temperature remains constant at 38.5°C during prolonged hot water immersion (Francisco et al., 2021). A longer duration to reach a higher rectal temperature may contribute to the differences observed in the study of Chiesa et al. (2016) compared to the present study where a 0.5°C greater increase in rectal temperature in the 40‐Shoulder condition did not result in difference in superficial femoral shear rate when compared to the 40‐Waist condition (Δ12/s) that had a similar increase in thigh skin temperature (Δ0.1°C). Indeed, a significant strength of the present work is the two duration‐matched conditions with similar increases in rectal temperature (i.e., 40‐Shoulder and 42‐Waist), which allow for the effects of rectal temperature to be examined without being confounded by differences in heating duration.

In a non‐submerged limb (e.g., the arms), elevations in rectal temperature during waist‐deep immersion, had a systemic effect resulting in increased brachial artery shear rate by 206/s and 314/s in the 40‐Waist and 42‐Waist conditions, respectively. These findings are in accordance with previous work, albeit with a longer immersions duration of 45 min, demonstrating that knee and ankle‐deep immersion increases brachial artery shear rate by ∼300–600/s, when accompanied by ∼0.5°C elevations in core body temperature (Cheng et al., 2021). The addition of muscle temperature measurements in the arms and legs would have provided a more comprehensive characterisation of peripheral temperature and enabled greater discussion of the effects of local versus systemic temperature elevation; however, these data clearly demonstrate a systemic effect of lower body heating.

### 4.2 Inflammatory responses

Plasma IL‐6 concentrations did not differ following 30 min of hot water immersion, whilst cortisol concentrations reduced. Both IL‐6 and cortisol concentrations have previously been shown to acutely increase following passive heating protocols (Hoekstra et al., 2018, 2021; Leicht et al., 2015; Mansfield et al., 2021; Steward et al., 2024). However, the duration of heating in these studies is at least 60 min compared to 30 min in the present study. Cortisol appears to have a biphasic response to heating, whereby there is an initial decrease in concentration followed by a subsequent increase with heating protocols longer than 30 min (Ježová et al., 1994; Leicht et al., 2015; Steward et al., 2024). With exercise, there is an exponential relationship between duration and changes in IL‐6 (Fischer, 2006), and although the cause of increases in IL‐6 concentration may differ between exercise and passive heating, a similar relationship may exist with passive heating. In support of this, observations from our laboratory have shown relatively small (∼0.3 pg/mL) increases in IL‐6 concentrations after 60 but not 30 min of arms in 40°C water immersion (Steward et al., 2024). Whilst IL‐6 did not show a significant effect of time in the present study, η_p_^2^ was 0.17, with a *P*‐value of 0.06, suggesting that the present data were potentially underpowered to show this effect. Regardless of this, the 0.11 ± 0.44 pg/mL increase in IL‐6 concentration observed in the present study is of smaller magnitude than previously observed with similar duration moderate‐intensity exercise (Δ∼0.2 pg/mL) (Cullen et al., 2016), or greater heating durations (Δ∼0.4–1.0 pg/mL) (Hoekstra et al., 2021; Mansfield et al., 2021; Steward et al., 2024). Importantly, the present study showed that alterations to the water temperature or depth of immersion did not alter the acute inflammatory response. Therefore, when considered in the context of the existing literature, duration appears to be a key factor in stimulating an acute inflammatory response to hot water immersion. Indeed, whilst repeated 30‐min heating bouts can elicit vascular or thermoregulatory adaptation (Bailey et al., 2016; Carter, Spence, Atkinson, Pugh, Naylor, et al., 2014), longer heating durations may be required to induce the IL‐6 signalling pathway, such as activation of anti‐inflammatory cytokines (e.g., IL‐10) and assist with the prevention of chronic inflammatory and metabolic diseases. However, longer exposure durations may come at the expense of enjoyment and tolerability.

### 4.3 Tolerability, enjoyment and adverse responses

The present study showed 30 min of immersion was tolerated by all participants in all conditions. However, conditions with larger increases in rectal temperature (40‐Shoulder and 42‐Waist), through increasing either immersion depth or water temperature, demonstrated poorer perceptual responses and a greater desire to exit. Moreover, 45% of participants reported mild–moderate symptoms of dizziness upon standing following immersion in these conditions. This reinforces the need for caution in naïve users of hot water immersion and the need for more research to identify predictors of standing dizziness. The 40‐Waist condition in the present study demonstrated the least negative perceptual responses. Whilst this may lead to longer self‐selected durations, or better adherence to a long‐term intervention as suggested by Hoekstra et al. (2021), our data show that these improved perceptual responses come at the cost of attenuated shear stress and thermal stimuli required for chronic adaptation. However, an unexpected observation was that thermal comfort improved rapidly at the end of immersion in the 40‐Shoulder condition when participants had moved onto the stool and removed their arms from the water. This may provide support for the implementation of an immersion protocol where immersion depth begins with maximal body coverage but is reduced after an initial period (e.g., as performed in Brunt et al. (2016) to extend the immersion duration without it becoming intolerable, thereby attempting to maximise the trade‐off between beneficial physiological and favourable perceptual responses.

### 4.4 Perspectives

The observed vascular effects in the present study have important practical implications for the design and application of hot water immersion protocols. This study showed that increases in shear rate are more responsive to changes in local temperature than core body temperature, meaning that limb submersion results in the largest effect on arterial haemodynamic responses, whilst increasing water temperature augments this response in both submerged and non‐submerged limbs. These vessel‐ and limb‐specific responses, mean that protocols described in insufficient detail (e.g., depth of immersion, immersion of limbs) may be at risk of inaccurate generalisations across vascular beds exposed to different local temperatures and erroneous interpretations of acute haemodynamic effects of a given protocol.

The acute responses shown in the present study are essential for long term adaptation with repeated exposures (Carter, Spence, Atkinson, Pugh, Naylor, et al., 2014; Dawson et al., 2018; Roxburgh et al., 2023) and suggest that for systemic adaptation, maximising immersion depth or surface area of the body heated is important for heating protocol selection, whilst local vascular adaptation may be better served through a hotter water temperature with a reduced immersion depth. The magnitude of these differences for shear rate was 81/s in the brachial artery and 59/s in the superficial femoral artery between the 40‐Shoulder and 42‐Waist conditions. Based on the correlations observed by Dawson et al. (2018), this would equate to a ∼2–4% greater increase in flow‐mediated dilatation if these stimuli were repeated as a measure of improved endothelial function and vascular health. Moreover, the present findings on limb/vascular bed‐specific responses may be relevant for individuals seeking vascular adaptation who may implement hot water immersion differently depending on the requirements of their health condition. For example, patients with claudication of the lower limbs, as seen in people with peripheral artery disease, may favour waist‐deep immersion in a hotter water temperature for increases in lower limb shear stress and blood flow, as previously investigated by Thomas et al. (2017). In contrast, in the general population brachial artery endothelial function is reflective of systemic vascular health (Broxterman et al., 2019), and therefore, a greater immersion depth may be more beneficial. This is also supported by the present blood pressure data with the greatest acute reduction in the 40‐Shoulder condition of –10 mmHg for DBP, which would equate to a chronic reduction of ∼−2 to 5 mmHg with repeated exposure based on the data observed by Roxburgh et al. (2023). Accordingly, these data may be useful to inform future interventional research and practical implementation; however, the present data were collected from young, healthy volunteers, and more research is required to investigate the potential different vascular adaptive effects of these immersion protocols in longitudinal interventions with different population groups.

### 4.5 Summary

This study provides a strong basis for elucidating different physiological responses to alterations in hot water immersion protocols and demonstrates the potential trade‐off between the thermal and arterial haemodynamic stimuli and perceptual responses. Increases in water temperature and immersion depth during 30‐min immersion protocols do not result in different effects on inflammation but have distinct region‐specific arterial haemodynamic effects. Beneficial physiological responses may be accompanied with less favourable perceptual responses, demonstrating a potential trade‐off that should be considered during both acute and chronic protocol implementation.

## AUTHOR CONTRIBUTIONS

Campbell Menzies, Tom Cullen, Neil Clarke, Christopher Pugh and C. Douglas Thake were responsible for the conception and design of the study. Campbell Menzies, Charles Steward and Tom Cullen were responsible for data acquisition, while all authors assisted in the interpretation of the data. All authors contributed to drafting or revision of the written work, approved the final version and agree to be accountable for all aspects of the work in ensuring that questions related to the accuracy or integrity of any part of the work are appropriately investigated and resolved. All persons designated as authors qualify for authorship, and all those who qualify for authorship are listed.

## CONFLICT OF INTEREST

None declared.

## FUNDING INFORMATION

None.

## Supporting information




**Supplementary Material**: eph13944s‐sup‐0001‐SuppMat.xlsx

**Supplementary Material**: eph13944s‐sup‐0002‐SuppMat.docx

## Data Availability

Data available in Supporting information.
