# Supplementary material for: Vascular, inflammatory and perceptual responses to hot water immersion: Impacts of water depth and temperature in young healthy adults
Source: Exp Physiol. 2025 Jul 29:10.1113/EP092761. Online ahead of print. doi: 10.1113/EP092761 (PMC13394111; doi:10.1113/EP092761)

# Supplementary Material

## Rcode for statistical analysis

#anova

aov_variable <- anova_test(

data = Data dv = Value,

wid = Participant,

within = c(Time,Condition),

type = 3,

effect.size = "pes")

aov_variable

Bonferroni_variable <- Data_variable %>%

group_by(Time) %>%

pairwise_t_test(

Value~Condition, paired = TRUE,

p.adjust.method = "bonferroni"

)

Bonferroni_variable

#perceptual data

#condition

`40_shoulder` <- Data %>%

subset(Condition == "40-Shoulder")

friedman.test(y = `40_shoulder`$Value, groups = `40_shoulder`$Time, blocks = `40_shoulder`$Participant)

`40_waist` <- Data%>%

subset(Condition == "40-Waist")

friedman.test(y = `40_waist`$Value, groups = `40_waist`$Time, blocks = `40_waist`$Participant)

`42_waist` <- Data %>%

subset(Condition == "42-Waist")

friedman.test(y = `42_waist`$Value, groups = `42_waist`$Time, blocks = `42_waist`$Participant)

#condition*time

Time_0<- Data_%>%

subset(Time == "0")

friedman.test(y = Time_0$Value, groups = Time_0$Condition, blocks = Time_0$Participant)

Time_10<- Data_ %>%

subset(Time == "10")

friedman.test(y = Time_10$Value, groups = Time_10$Condition, blocks = Time_10$Participant)

pairwise.wilcox.test(Time_10$Value, Time_10$Condition, p.adj = "bonf")

Time_20<- Data%>%

subset(Time == "20")

friedman.test(y = Time_20$Value, groups = Time_20$Condition, blocks = Time_20$Participant)

pairwise.wilcox.test(Time_20$Value, Time_20$Condition, p.adj = "bonf")

Time_30<- Data %>%

subset(Time == "30")

#preferences questionnaire

friedman.test(y = Data_pref$Value, groups = Data_pref$Condition, blocks = Data_pref$Participant)

#desired exit time

Exit_30 <- Exit_time %>%

subset(Time == 30 & Exit == "Y")

M <- table(Exit_30$Condition, Exit_30$Value)

chisq.test(Exit_30$Condition, Exit_30$Value)

library("remotes")

install_version("fifer", "1.0")

# Import

library("fifer")

# Run Post Hoc Test of Proportion

chisq.post.hoc(M, control = "bonferroni")

#exit 15

Exit_15 <- Exit_time %>%

subset(Time == 15) %>%

subset(Exit =="Y")

M <- table(Exit_15$Condition, Exit_15$Value)

fisher.test(M)

chisq.test(Exit_15$Condition, Exit_15$Value)

chisq.post.hoc(M, control = "bonferroni")

#exit 20

Exit_20 <- Exit_time %>%

subset(Time == 20) %>%

subset(Exit =="Y")

M <- table(Exit_20$Condition, Exit_20$Value)

chisq.test(Exit_20$Condition, Exit_20$Value)

fisher.test(M)

chisq.post.hoc(M, control = "bonferroni", test = "fisher.test")

#exit 25

Exit_25 <- Exit_time %>%

subset(Time == 25) %>%

subset(Exit =="Y")

M <- table(Exit_25$Condition, Exit_25$Value)

chisq.test(Exit_25$Condition, Exit_25$Value)

fisher.test(M)

chisq.post.hoc(M, control = "bonferroni", test = "fisher.test")

## Sex differences

Data analysed using the following code:

anova_test( data = Data, dv = Value, wid = Participant, within = c(Time,Condition), between = Sex, type = 3, effect.size = "pes")

Data %>% group_by(Time, Condition) %>% pairwise_t_test( Value~Sex, paired = FALSE, p.adjust.method = "bonferroni")

1. Rectal temperature

| Condition | Sex | 0 | 5 | 10 | 15 | 20 | 25 | 30 | 40 |
| --- | --- | --- | --- | --- | --- | --- | --- | --- | --- |
| 40-Shoulder | F | 37.0 (0.3) | 37.0 (0.3) | 37.0 (0.3) | 37.2 (0.3) | 37.4 (0.3) | 37.8 (0.3) | 37.9 (0.3) | 37.8 (0.3) |
| 40-Shoulder | M | 36.8 (0.3) | 36.9 (0.3) | 37.0 (0.3) | 37.2 (0.3) | 37.3 (0.3) | 37.6 (0.3) | 37.8 (0.3) | 37.6 (0.3) |
| 40-Waist | F | 37.0 (0.3) | 37.1 (0.3) | 37.2 (0.3) | 37.2 (0.3) | 37.3 (0.3) | 37.4 (0.3) | 37.6 (0.3) | 37.5 (0.3) |
| 40-Waist | M | 36.8 (0.2) | 36.9 (0.2) | 36.9 (0.2) | 37.0 (0.2) | 37.1 (0.2) | 37.2 (0.2) | 37.3 (0.2) | 37.3 (0.2) |
| 42-Waist | F | 37.1 (0.2) | 37.2 (0.2) | 37.3 (0.2) | 37.5 (0.3) | 37.7 (0.3) | 37.9 (0.3) | 38.1 (0.3) | 37.7 (0.2) |
| 42-Waist | M | 36.8 (0.1) | 36.9 (0.1) | 37.1 (0.1) | 37.3 (0.2) | 37.4 (0.2) | 37.6 (0.2) | 37.7 (0.2) | 37.6 (0.2) |


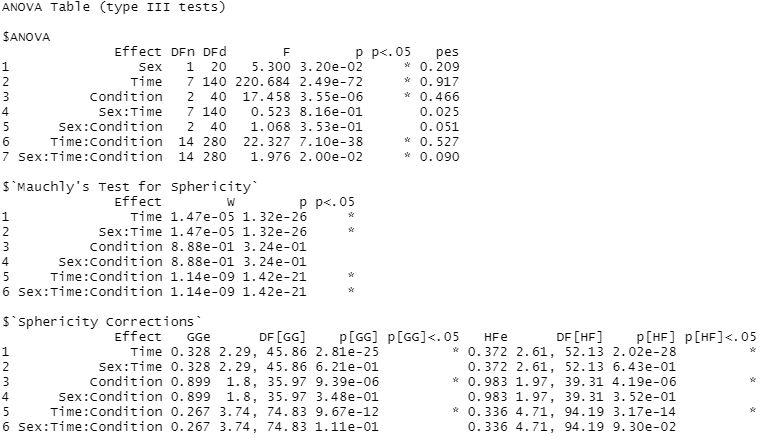


1. Heart rate

| Condition | Sex | 0 | 5 | 10 | 15 | 20 | 25 | 30 |
| --- | --- | --- | --- | --- | --- | --- | --- | --- |
| 40-Shoulder | F | 57 (13) | 64 (13) | 74 (16) | 84 (20) | 88 (19) | 93 (19) | 93 (16) |
| 40-Shoulder | M | 53 (10) | 67 (11) | 75 (13) | 83 (13) | 90 (13) | 96 (16) | 97 (15) |
| 40-Waist | F | 58 (9) | 67 (11) | 72 (12) | 74 (14) | 83 (16) | 87 (15) | 89 (13) |
| 40-Waist | M | 55 (11) | 67 (12) | 73 (11) | 77 (13) | 84 (14) | 87 (13) | 87 (15) |
| 42-Waist | F | 59 (11) | 75 (16) | 84 (16) | 91 (15) | 99 (19) | 101 (19) | 106 (18) |
| 42-Waist | M | 55 (11) | 78 (17) | 87 (17) | 96 (20) | 99 (22) | 101 (19) | 105 (18) |


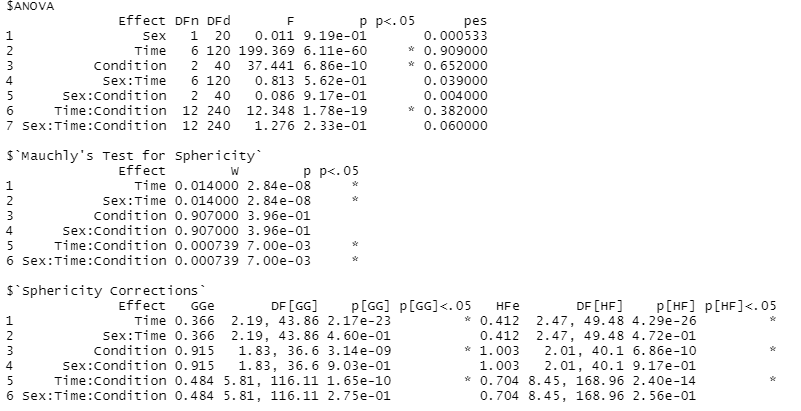


1. Nude mass

| Condition | Sex | diff |
| --- | --- | --- |
| 40-Shoulder | F | -0.4 (0.1) |
| 40-Shoulder | M | -0.5 (0.2) |
| 40-Waist | F | -0.2 (0.1) |
| 40-Waist | M | -0.3 (0.2) |
| 42-Waist | F | -0.3 (0.1) |
| 42-Waist | M | -0.5 (0.3) |


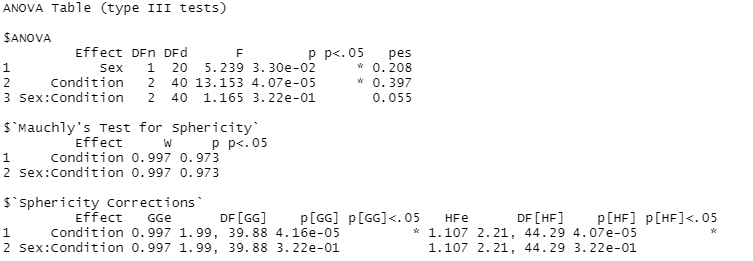


1. SBP

| Condition | Sex | Pre | 5 | 15 | 25 | Post |
| --- | --- | --- | --- | --- | --- | --- |
| 40-Shoulder | F | 111 (7) | 99 (8) | 102 (9) | 104 (9) | 114 (7) |
| 40-Shoulder | M | 123 (9) | 116 (10) | 118 (13) | 119 (11) | 128 (7) |
| 40-Waist | F | 112 (9) | 94 (12) | 95 (9) | 94 (11) | 114 (8) |
| 40-Waist | M | 124 (9) | 112 (11) | 116 (16) | 117 (14) | 129 (9) |
| 42-Waist | F | 111 (6) | 97 (8) | 98 (9) | 104 (11) | 118 (7) |
| 42-Waist | M | 122 (7) | 114 (14) | 121 (13) | 120 (12) | 128 (6) |


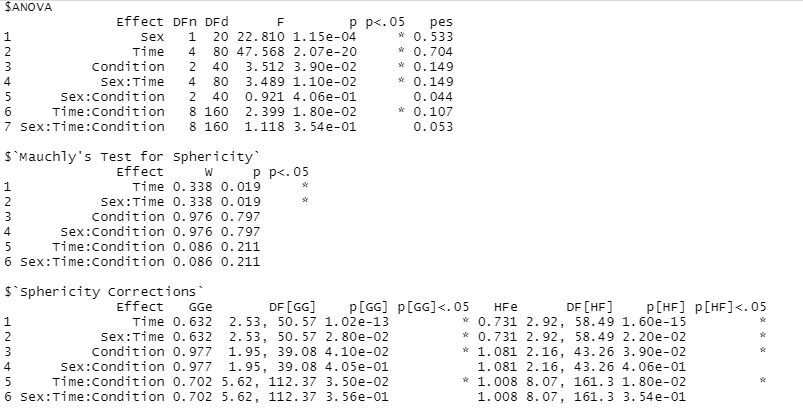


1. DBP

| Condition | Sex | Pre | 5 | 15 | 25 | Post |
| --- | --- | --- | --- | --- | --- | --- |
| 40-Shoulder | F | 67 (7) | 56 (6) | 52 (9) | 50 (6) | 54 (9) |
| 40-Shoulder | M | 69 (6) | 64 (8) | 59 (9) | 57 (7) | 61 (8) |
| 40-Waist | F | 71 (6) | 56 (5) | 58 (10) | 52 (5) | 66 (6) |
| 40-Waist | M | 70 (6) | 61 (8) | 62 (9) | 63 (8) | 67 (7) |
| 42-Waist | F | 69 (6) | 58 (12) | 51 (7) | 52 (8) | 57 (9) |
| 42-Waist | M | 70 (4) | 62 (7) | 63 (8) | 61 (7) | 65 (8) |


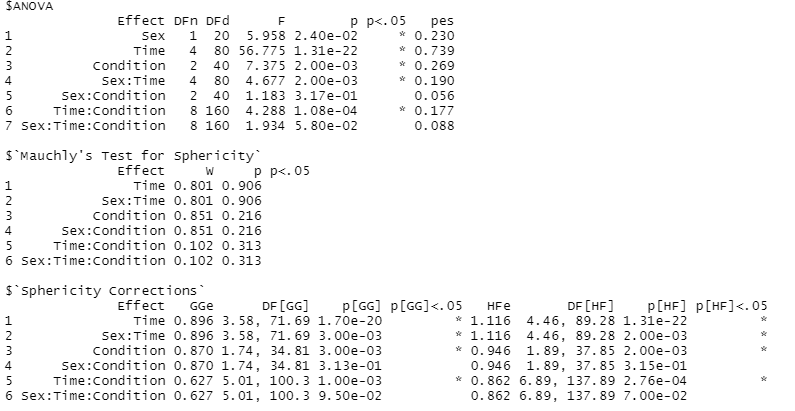


1. Dizziness upon standing

| Condition | Sex | Rest | Post |
| --- | --- | --- | --- |
| 40-Shoulder | F | 0 (1) | 1 (2) |
| 40-Shoulder | M | 0 (0) | 3 (3) |
| 40-Waist | F | 0 (0) | 0 (1) |
| 40-Waist | M | 0 (0) | 1 (2) |
| 42-Waist | F | 0 (0) | 1 (2) |
| 42-Waist | M | 0 (0) | 2 (2) |


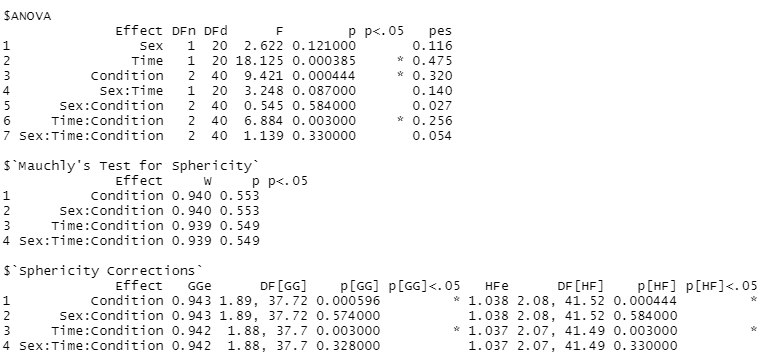


1. Maximum heart rate upon standing

| Condition | Sex | Rest | Post |
| --- | --- | --- | --- |
| 40-Shoulder | F | 77 (13) | 134 (19) |
| 40-Shoulder | M | 78 (10) | 130 (13) |
| 40-Waist | F | 80 (9) | 118 (15) |
| 40-Waist | M | 79 (10) | 111 (13) |
| 42-Waist | F | 78 (11) | 137 (18) |
| 42-Waist | M | 79 (13) | 129 (16) |


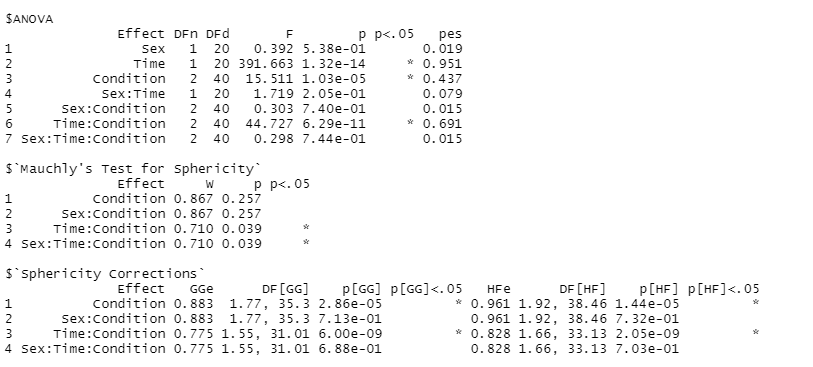


1. Sit-stand SBP

| Condition | Sex | Rest | Post |
| --- | --- | --- | --- |
| 40-Shoulder | F | 4 (6) | -8 (12) |
| 40-Shoulder | M | 4 (5) | -15 (13) |
| 40-Waist | F | 8 (10) | 3 (15) |
| 40-Waist | M | 13 (34) | 4 (37) |
| 42-Waist | F | 5 (4) | 2 (10) |
| 42-Waist | M | 12 (37) | -7 (11) |


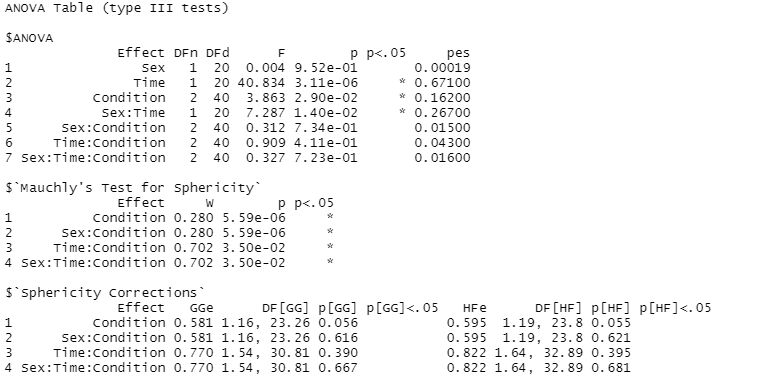


1. Sit-stand DBP

| Condition | Sex | Rest | Post |
| --- | --- | --- | --- |
| 40-Shoulder | F | 3 (4) | -16 (8) |
| 40-Shoulder | M | 2 (5) | -16 (6) |
| 40-Waist | F | 7 (10) | 2 (16) |
| 40-Waist | M | 7 (24) | 0 (25) |
| 42-Waist | F | 3 (3) | -8 (9) |
| 42-Waist | M | 6 (18) | -8 (6) |


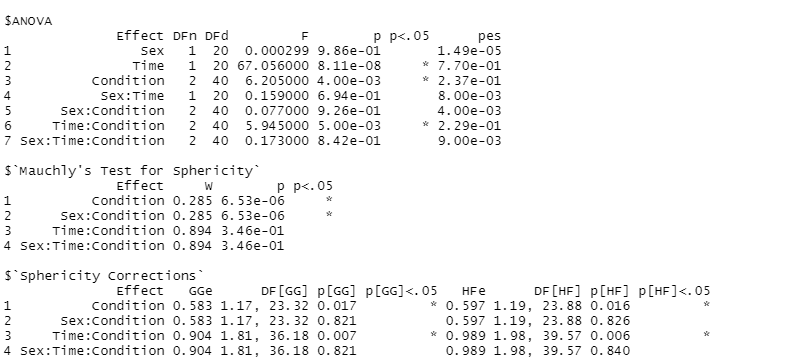


1. Femoral diameter

| Condition | Sex | Pre | Post |
| --- | --- | --- | --- |
| 40-Shoulder | F | 0.55 (0.06) | 0.60 (0.06) |
| 40-Shoulder | M | 0.65 (0.07) | 0.69 (0.08) |
| 40-Waist | F | 0.52 (0.07) | 0.58 (0.06) |
| 40-Waist | M | 0.65 (0.09) | 0.68 (0.08) |
| 42-Waist | F | 0.54 (0.07) | 0.60 (0.07) |
| 42-Waist | M | 0.65 (0.09) | 0.69 (0.09) |


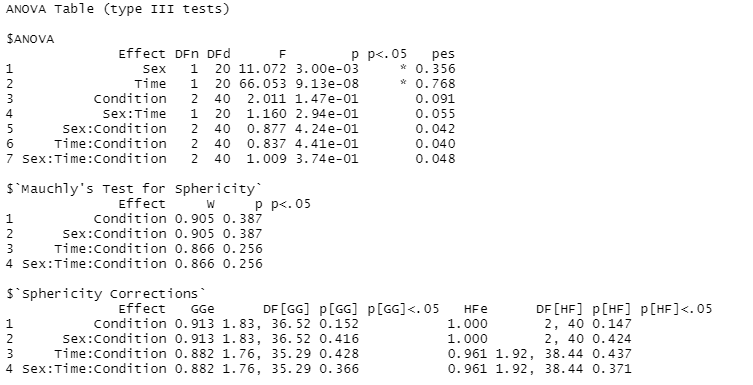


1. Femoral mean shear rate

| Condition | Sex | Pre | Post |
| --- | --- | --- | --- |
| 40-Shoulder | F | 52.6 (16.9) | 238.9 (52.9) |
| 40-Shoulder | M | 44.1 (24.3) | 157.8 (50.8) |
| 40-Waist | F | 92.0 (83.1) | 266.1 (93.7) |
| 40-Waist | M | 44.2 (23.2) | 144.6 (36.5) |
| 42-Waist | F | 67.7 (29.8) | 331.3 (90.0) |
| 42-Waist | M | 46.7 (22.3) | 196.1 (59.5) |


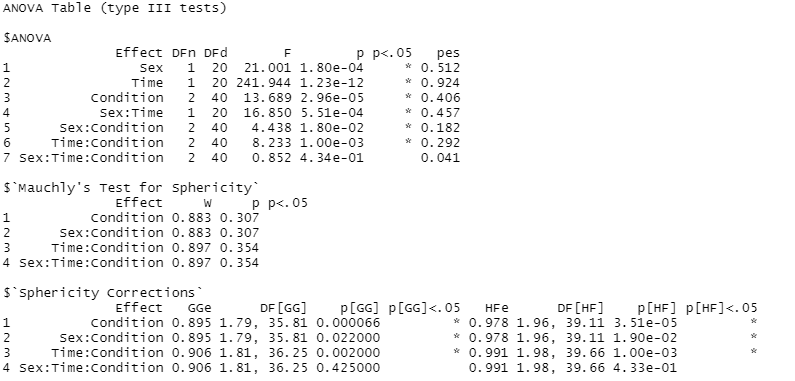


1. Femoral mean blood flow

| Condition | Sex | Pre | Post |
| --- | --- | --- | --- |
| 40-Shoulder | F | 112 (39) | 606 (158) |
| 40-Shoulder | M | 141 (61) | 624 (187) |
| 40-Waist | F | 142 (78) | 613 (254) |
| 40-Waist | M | 141 (53) | 545 (170) |
| 42-Waist | F | 126 (36) | 868 (278) |
| 42-Waist | M | 151 (63) | 769 (249) |


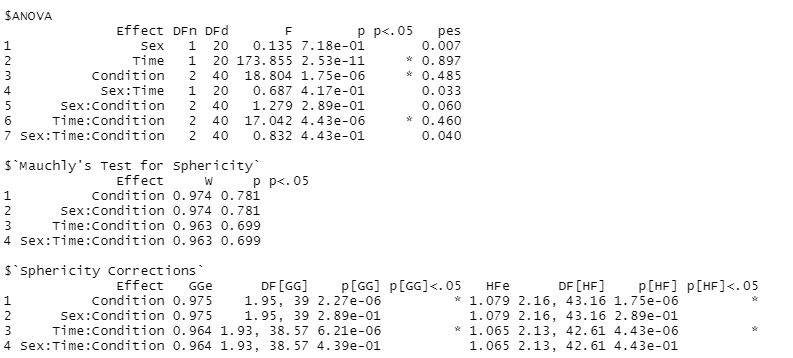


1. Femoral antegrade shear rate

| Condition | Sex | Pre | Post |
| --- | --- | --- | --- |
| 40-Shoulder | F | 83 (19) | 241 (49) |
| 40-Shoulder | M | 73 (30) | 164 (46) |
| 40-Waist | F | 119 (75) | 268 (91) |
| 40-Waist | M | 72 (27) | 153 (33) |
| 42-Waist | F | 101 (34) | 331 (90) |
| 42-Waist | M | 72 (27) | 202 (54) |


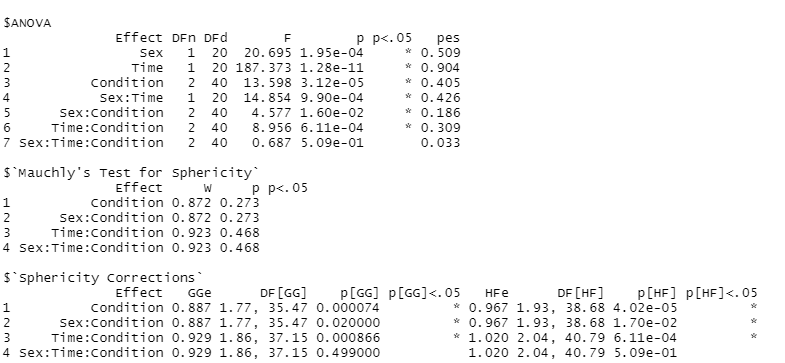


1. Femoral retrograde shear rate

| Condition | Sex | Pre | Post |
| --- | --- | --- | --- |
| 40-Shoulder | F | -31 (9) | -2 (5) |
| 40-Shoulder | M | -29 (14) | -6 (8) |
| 40-Waist | F | -27 (17) | -2 (5) |
| 40-Waist | M | -28 (13) | -8 (7) |
| 42-Waist | F | -33 (12) | 0 (1) |
| 42-Waist | M | -25 (12) | -5 (8) |


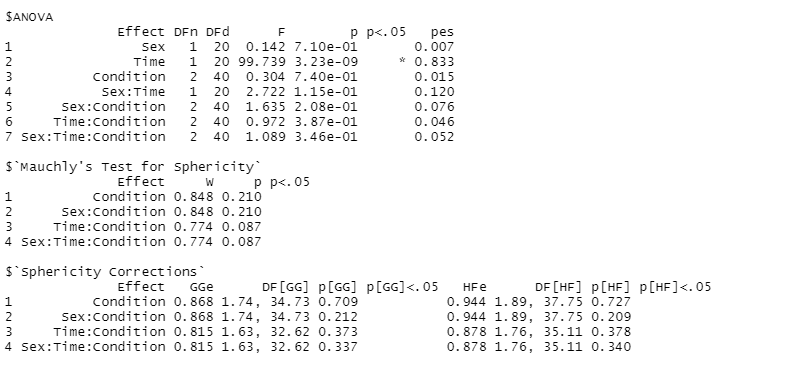


1. Brachial diameter

| Condition | Sex | Pre | 5 | 15 | 25 | Post |
| --- | --- | --- | --- | --- | --- | --- |
| 40-Shoulder | F | 0.32 (0.05) | 0.30 (0.06) | 0.34 (0.05) | 0.36 (0.06) | 0.35 (0.06) |
| 40-Shoulder | M | 0.42 (0.04) | 0.43 (0.04) | 0.46 (0.05) | 0.48 (0.04) | 0.47 (0.04) |
| 40-Waist | F | 0.30 (0.06) | 0.30 (0.04) | 0.30 (0.04) | 0.31 (0.05) | 0.30 (0.04) |
| 40-Waist | M | 0.42 (0.04) | 0.41 (0.04) | 0.42 (0.03) | 0.42 (0.04) | 0.41 (0.05) |
| 42-Waist | F | 0.30 (0.05) | 0.29 (0.05) | 0.31 (0.05) | 0.32 (0.07) | 0.31 (0.05) |
| 42-Waist | M | 0.42 (0.04) | 0.41 (0.04) | 0.42 (0.04) | 0.44 (0.04) | 0.43 (0.05) |


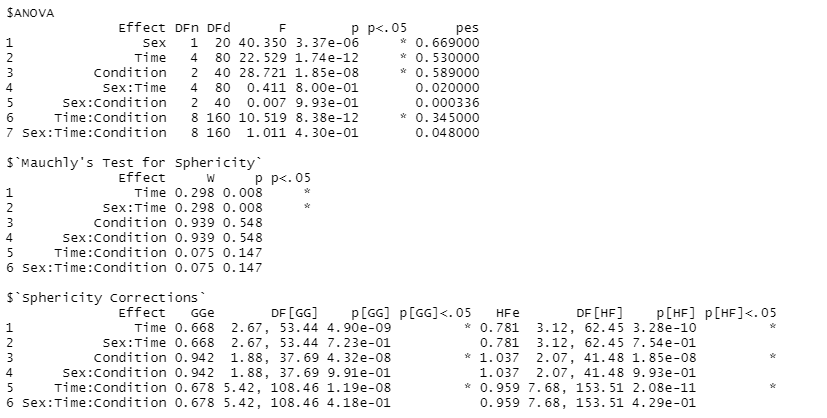


1. Brachial mean shear rate

| Condition | Sex | Pre | 5 | 15 | 25 | Post |
| --- | --- | --- | --- | --- | --- | --- |
| 40-Shoulder | F | 42 (20) | 163 (82) | 474 (132) | 549 (119) | 183 (110) |
| 40-Shoulder | M | 44 (38) | 172 (71) | 339 (130) | 390 (118) | 151 (65) |
| 40-Waist | F | 44 (18) | 132 (112) | 186 (94) | 307 (142) | 38 (32) |
| 40-Waist | M | 64 (51) | 88 (55) | 165 (70) | 230 (72) | 32 (24) |
| 42-Waist | F | 47 (26) | 141 (109) | 300 (114) | 402 (161) | 96 (88) |
| 42-Waist | M | 56 (42) | 128 (84) | 239 (89) | 342 (110) | 102 (57) |


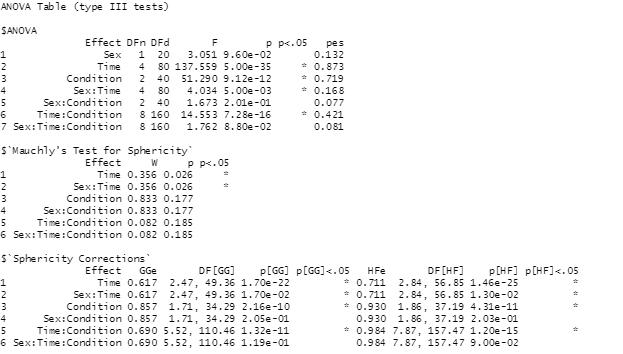


1. Brachial mean blood flow

| Condition | Sex | Pre | 5 | 15 | 25 | Post |
| --- | --- | --- | --- | --- | --- | --- |
| 40-Shoulder | F | 15 (6) | 53 (29) | 238 (127) | 341 (181) | 107 (70) |
| 40-Shoulder | M | 38 (33) | 152 (54) | 378 (128) | 491 (120) | 189 (100) |
| 40-Waist | F | 15 (8) | 41 (33) | 68 (51) | 124 (86) | 13 (12) |
| 40-Waist | M | 56 (49) | 71 (39) | 148 (67) | 215 (92) | 30 (24) |
| 42-Waist | F | 16 (10) | 54 (56) | 129 (83) | 206 (146) | 50 (56) |
| 42-Waist | M | 50 (40) | 105 (67) | 218 (90) | 339 (108) | 112 (82) |


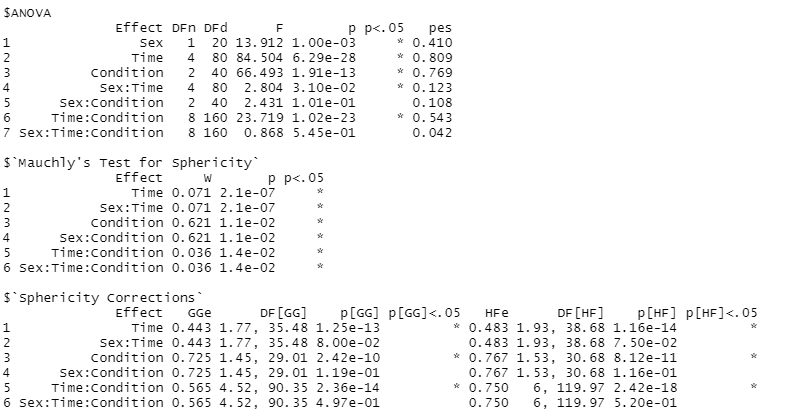


1. Brachial antegrade

| Condition | Sex | Pre | 5 | 15 | 25 | Post |
| --- | --- | --- | --- | --- | --- | --- |
| 40-Shoulder | F | 59 (25) | 191 (75) | 474 (131) | 549 (118) | 184 (110) |
| 40-Shoulder | M | 59 (41) | 179 (67) | 340 (128) | 393 (116) | 151 (65) |
| 40-Waist | F | 57 (22) | 175 (94) | 222 (87) | 325 (129) | 64 (34) |
| 40-Waist | M | 76 (50) | 116 (51) | 186 (61) | 240 (64) | 42 (22) |
| 42-Waist | F | 62 (33) | 186 (83) | 311 (105) | 415 (138) | 107 (83) |
| 42-Waist | M | 65 (42) | 153 (71) | 252 (82) | 349 (104) | 108 (56) |


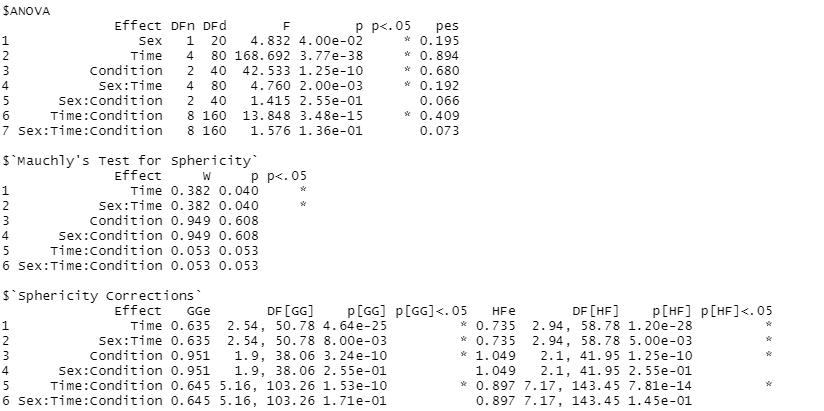


1. Brachial retrograde

| Condition | Sex | Pre | 5 | 15 | 25 | Post |
| --- | --- | --- | --- | --- | --- | --- |
| 40-Shoulder | F | -17 (11) | -28 (24) | 0 (1) | 0 (0) | 0 (1) |
| 40-Shoulder | M | -15 (16) | -7 (9) | -2 (6) | -3 (5) | 0 (0) |
| 40-Waist | F | -14 (7) | -43 (41) | -36 (48) | -18 (29) | -26 (13) |
| 40-Waist | M | -12 (12) | -28 (18) | -22 (25) | -10 (12) | -10 (10) |
| 42-Waist | F | -15 (10) | -45 (37) | -11 (13) | -13 (30) | -11 (11) |
| 42-Waist | M | -9 (9) | -26 (26) | -13 (13) | -7 (9) | -6 (6) |


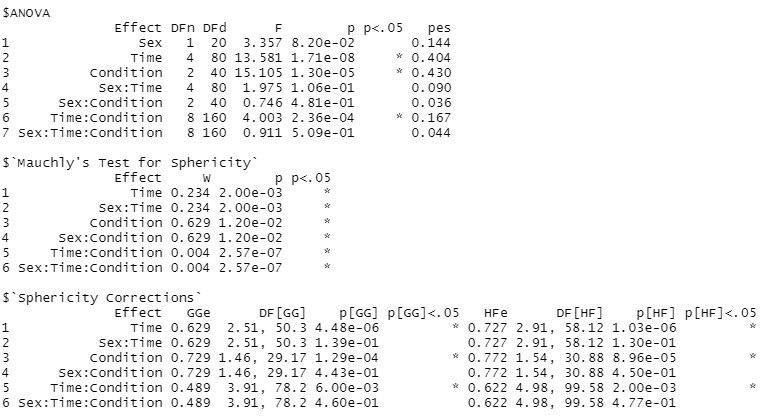


1. IL6

| Condition | Sex | Pre | Post |
| --- | --- | --- | --- |
| 40-Shoulder | F | 0.79 (0.62) | 0.87 (0.66) |
| 40-Shoulder | M | 1.00 (0.55) | 1.19 (0.81) |
| 40-Waist | F | 0.94 (0.40) | 0.97 (0.51) |
| 40-Waist | M | 1.06 (0.59) | 1.25 (0.90) |
| 42-Waist | F | 0.87 (0.49) | 1.01 (0.76) |
| 42-Waist | M | 0.84 (0.69) | 0.83 (0.35) |


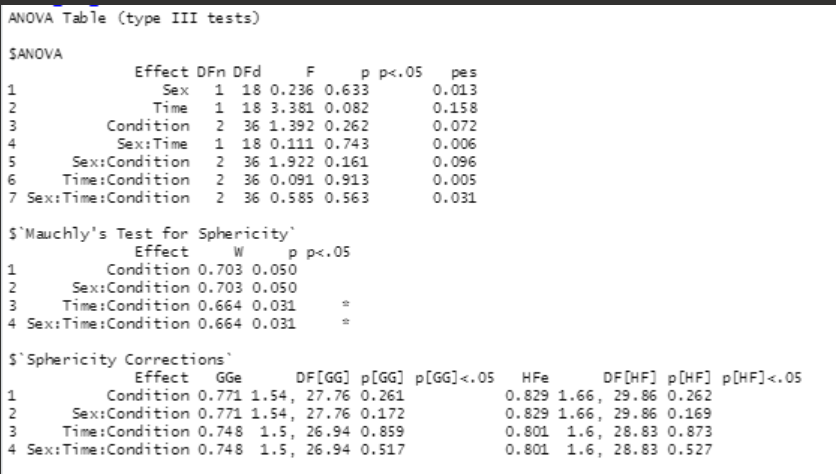


1. Cortisol

| Condition | Sex | Pre | Post |
| --- | --- | --- | --- |
| 40-Shoulder | F | 95 (24) | 60 (26) |
| 40-Shoulder | M | 76 (28) | 53 (29) |
| 40-Waist | F | 92 (32) | 50 (12) |
| 40-Waist | M | 84 (38) | 60 (19) |
| 42-Waist | F | 80 (28) | 57 (24) |
| 42-Waist | M | 87 (41) | 61 (29) |


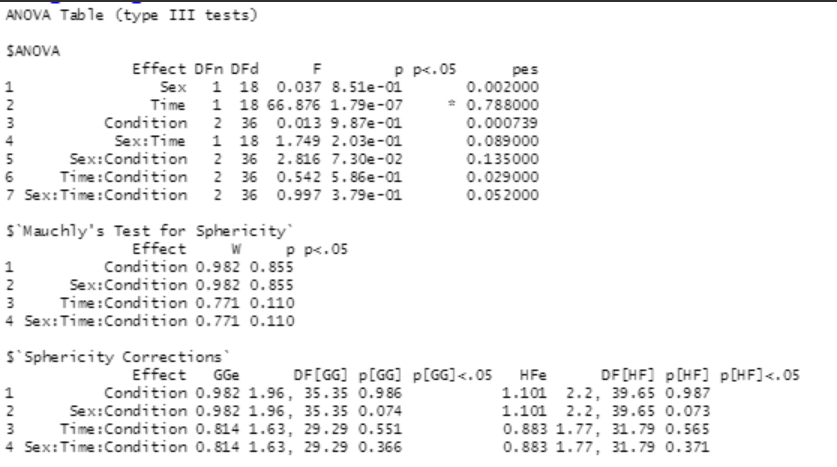

Supplement: Supplementary file 2 — Supplementary Material: eph13944s‐sup‐0002‐SuppMat.docx [file EPH-9999-0-s002.docx]
